# 666 Implementation of a Nurse-Led Program to Improve Burn and Trauma Outcomes

**DOI:** 10.1093/jbcr/iraf019.295

**Published:** 2025-04-01

**Authors:** Mack Drake, Jeffrey Litt, Jessica Brady, Rachel Norton, Hali Wolf, Stanley Kurek

**Affiliations:** Burn and Reconstructive Center at Chippenham Hospital; Burn and Reconstructive Centers of Virginia Chippenham; Chippenham Hospital; Burn and Reconstructive Centers of Virginia Chippenham; Burn and Reconstructive Centers of Virginia Chippenham; Burn and Reconstructive Centers of Virginia Chippenham

## Abstract

**Introduction:**

The Emergency Department is the critical entry point for burn patients requiring immediate and complex care. To enhance patient outcomes, our regional referral level 1 burn and trauma center created and implemented a Trauma Nurse Lead (TNL) position. This role focuses on optimizing burn and trauma processes by coordinating a multi-disciplinary approach with evidence-based practices to ensure safe, efficient, and effective patient care. A novel training program with burn, trauma, and critical care experience was developed. We hypothesize that implementation of this specialized role will impact mortality.

**Methods:**

After IRB exemption, a retrospective review of our burn and trauma regulatory database was undertaken from 2019-2023. Burn and trauma patients were separated into pre- and post-implementation groups corresponding with TNL start in May, 2022. Demographics, injury characteristics, and hospital quality metrics were analyzed. Statistical analysis was performed using Fisher’s Exact test and Mann-Whitney U Test as appropriate.

**Results:**

58 burn activations were recorded in the pre-TNL group vs 226 in the post-TNL group and 2943 trauma pre- vs 6169 in the post. As a whole, patients were mostly male, white, and had an average age of 44. Mean Injury Severity Score was 10.97 pre- vs 6.5 post. We noted a significant difference in mortality during the pre- 6.12%, vs the post, 2.75% (p< 0.0001) lending mortality benefit paralleled with TNL implementation across the combined group. There was a statistically significant decrease in HLOS in the post- (5.51 days vs 4.85 p< 0.0001). Subgroup analysis focused on burn activations yielded 58 pre- vs 226 post-TNL. Most burn patients discharged home or to outpatient. Burn mortality decreased non-significantly after implementation (1.72% vs 0.89% p=0.3911). Mean EDLOS was significantly higher post-implementation (454.2 min vs 191.9 p=0.0002). There was no meaningful difference in time to OR for burn patients between the groups (mean 28.7 hours). There was a statistically significant increase in mean HLOS for burn patients in the post-implementation period (1.72 days vs 3.64 p=0.0158).

**Conclusions:**

Creation and implementation of a highly specialized TNL position and training program paralleled a significant improvement in mortality across both trauma and burn activated patients and a non-significant decrease in mortality in the burn activation subgroup. HLOS as a whole improved. Longer HLOS and ED LOS in the burn subgroup post-TNL implementation coincides with and reflects formal burn center designation and a subsequent increase in burn volume during that period. Mean time to OR for burn patients of 28.7 hours is well under the acceptable threshold of most centers. This study is limited in its retrospective nature and time period coinciding with burn growth.

**Applicability of Research to Practice:**

In the future we will continue to track the impact on patient safety and burn-specific quality outcomes afforded by this position.

**Funding for the Study:**

N/A

